# How the quality of GWAS of human lifespan and health span can be improved

**DOI:** 10.3389/fgene.2013.00125

**Published:** 2013-06-28

**Authors:** Anatoliy I. Yashin, Konstantin G. Arbeev, Deqing Wu, Liubov S. Arbeeva, Alexander M. Kulminski, Igor Akushevich, Irina Culminskaya, Eric Stallard, Svetlana V. Ukraintseva

**Affiliations:** Center for Population Health and Aging, Duke University Population Research Institute, Social Science Research Institute, Duke University, Durham, NC, USA

## Introduction

Most connections between phenotype and genetic variants detected in genome wide association studies (GWAS) of human longevity-related traits did not reach the genome-wide level of statistical significance. These estimates also suffer from the lack of replication of research findings in studies of independent populations (Deelen et al., 2011; Nebel et al., 2011). Critical analysis of these studies reveals underused reserves in the data that may improve the accuracy of genetic estimates. One such reserve deals with the proper use of genetic information contained in the age structure of study participants at the time of bio-specimen collection. As we will see later, this information has been ignored in genetic analyses of follow-up data.

In this paper, we explain how such information can be used in analyses of follow up data and elucidate the benefits of joint analyses of both types of data. The proposed approach exploits the fact that participants in prospective studies often have different ages at baseline. The bio-specimen collection is often (but not always) done at baseline. Studies included in the CHARGE Consortium [except for the Original cohort of the Framingham Heart Study (FHS) where the bio-specimen collection was done some time after baseline] are typical examples. Since data on lifespan or other durations are often incomplete (e.g., censored), the Cox's type regression models is usually implemented in GWAS of these data (where, in addition to other covariates, conditioning on age at baseline is used). Note that using follow up data alone and conditioning on age at baseline may be required by the goal of the study (e.g., in the search for genes responsible for longest survival after reaching certain age, e.g., after 95 or 100 years). However, conditioning on the ages at bio-specimen collection when some of these ages are high enough may diminish or even totally exclude a substantial part of the genetic variation in longevity, i.e., eliminate the effects we are looking for. This is because the oldest old individuals participating in bio-specimen collection are precisely those who passed the process of mortality selection in the genetically heterogeneous population, and, therefore, are likely to carry genetic variants linked with “longevity” alleles (assuming that such alleles exist). Thus conditioning on the age at genotyping, especially when the oldest old study participants are in the sample, may leave little hope that associations of remaining genetic variants with human longevity will reach genome-wide significance.

Such conditioning, however, seems to be a common practice in GWAS of human longevity-related traits dealing with prospective data. For example, a large group of researchers performed comprehensive genetic analyses of human lifespan and free of major diseases lifespan using data from nine studies of the CHARGE Consortium (Walter et al., 2011). The authors “conducted a survival analysis, adjusted for age at baseline and sex, to model continuous time to death or end of follow-up” [Walter et al. (2011), section “Methods”]. The Cox proportional hazards model was used to describe the connections between genetic variants and time to event.

Note that, in addition to eliminating useful associations, adjusting for age at baseline may produce a bias in the analyses if the bio-specimen collection has been performed well after the time of the first examination (baseline). Such a situation characterizes the FHS data, which is the part of the CHARGE Consortium.

It turns out, however, that in cases when the ages at bio-specimen collection include young adults and the oldest old individuals, the additional information about the role of genetic variants in lifespan can be obtained from the age patterns of genetic frequencies evaluated for any genetic variant even without using the follow up data. The approach based on comparison of genetic frequencies among individuals of different age categories is typically used in genetic studies of centenarians (Weir, 1996; Yashin et al., 1999, 2000; Tan et al., 2004). A monotonic increase in genetic frequency with age indicates that the corresponding variant is associated with lifespan increase (“longevity” allele). A monotonic decline in such frequency indicates that this variant contributes to shortening lifespan (deleterious, or “frailty” allele). In cases when genotyping involves individuals from a large spectrum of ages including young adults, old, and oldest old ages, there is an additional opportunity to improve the quality of genetic analyses which typically is not used in genetic association studies of follow up data. To realize such potential, new methods of association analyses are required.

## The approach to improve the quality of GWAS of human longevity

One such method for joint analyses of the two types of data is based on maximizing the joint likelihood function of the combined data comprised of the cross-sectional age patterns and follow-up data. This method is a special case of a more general approach (Arbeev et al., 2011) that also includes follow-up data on non-genotyped individuals which can also be added to the likelihood function whenever such information is available.

The total likelihood of the data is the product of the two likelihood functions representing each subset of available data. The benefits of such analyses are that both likelihoods are functions of the same parameters describing the relationship between genetic factors and the phenotype of interest. The total likelihood is a function of the mortality rates for carriers, μ(*x*|*G* = 1), and non-carriers, μ(*x*|*G* = 0), and the initial proportion of the index variant, *P*_0_ = *P*(*G* = 1) (see Arbeev et al., 2011). By maximizing this likelihood function, one can estimate the respective quantities and test the null hypothesis on coincidence of survival functions for carriers and non-carriers of the minor allele using the likelihood ratio test. The nature of the effect of such genetic variants on survival (e.g., the protective effect so that the survival curve for carriers is shifted to the right compared to non-carriers, or the deleterious one so that the curve for carriers is shifted to the left, or the trade-off so that survival curves intersect) can be understood by inspecting the respective estimates of parameters and/or visualizing the estimated survival curves.

## Example

We performed a simulation study to illustrate how the approach combining information on follow-up and information on ages at biospecimen collection improves the accuracy and power of parameter estimates compared to the analyses that use only data on follow-up. To make the simulations close to reality, we generated data structure resembling the Long Life Family Study (LLFS) data (see description, e.g., in Yashin et al., 2010). This study deals with follow-up data on mortality, involves GWAS, and has a relatively short follow-up period.

We assumed that carriers and non-carriers of some hypothetical allele in a population have mortality rates μ(*x*|*G*) = μ_0_(*x*)*e*^γ *G*^, where *G* = 0 for non-carriers and *G* = 1 for carriers, and the baseline mortality μ_0_(*x*) is the Gompertz function, i.e., ln μ_0_(*x*) = ln *a* + *bx*. The Gompertz parameters *a* and *b* correspond to the initial mortality at birth and the rate of exponential growth in mortality with age in non-carriers. The regression parameter γ modifies the initial mortality for carriers so that it becomes *ae*^γ^. We used the Gompertz parameters ln *a* = −9.0 and *b* = 0.08 to produce reasonable survival patterns corresponding to human populations, and the proportion of carriers at birth *P*_0_ = 0.25. The parameter γ varied from −0.5 to 0.5 with the interval 0.05 to simulate scenarios with different effect sizes.

First, we generated a large (10,000,000 individuals) “general population” assigning the genetic status (carrier/non-carrier of a hypothetical allele) to individuals in the general population in accordance with the initial proportion *P*_0_. Then we generated life spans for all these individuals from the respective probability distributions [i.e., those corresponding to the hazard μ_0_(*x*)*e*^γ *G*^ for carriers and μ_0_(*x*) for non-carriers, with the parameters defined above]. Then we assigned the hypothetical “age at entry” into the study to each individual in the population generated as a discrete random variable uniformly distributed over the interval 40–100 years. Such “ages at entry” were assumed to be the same as age at biospecimen collection, in line with the LLFS design. We collected a sample of 4500 individuals (close to the actual number of genotyped individuals in the LLFS data) whose life spans exceeded their hypothetical “age at entry.” Individuals with simulated life spans exceeding “age at entry” plus 6 years were considered censored at that age.

The above procedure was repeated 1000 times (in each scenario with respective γ) to generate 1000 datasets which were subsequently estimated using the likelihood that uses only follow-up information and the likelihood that takes into account information on ages at biospecimen collection in addition to follow-up data.

**Figure 1:**
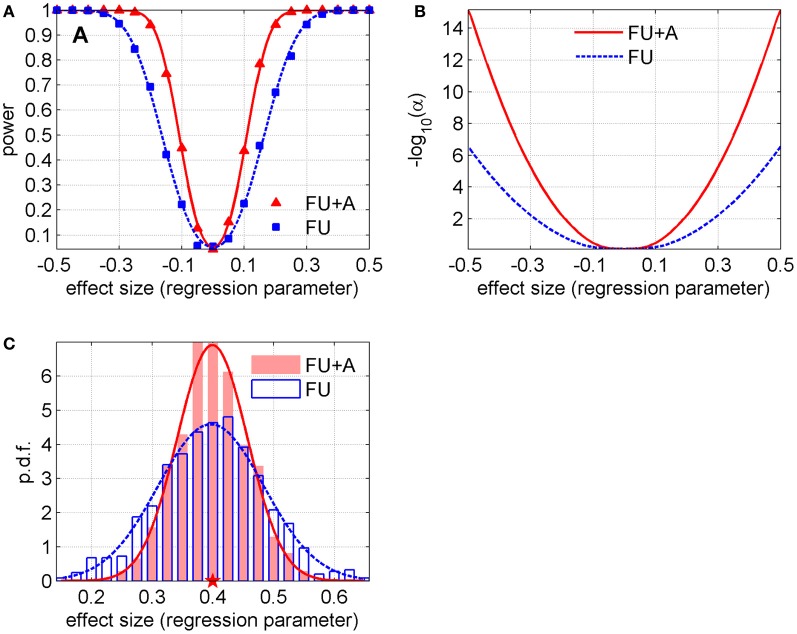
**(A)** Power in two methods described in the text (with follow-up only, “FU,” and follow-up and ages at biospecimen collection, “FU + A”) for different effect sizes (i.e., values of the regression parameter γ) and fixed α = 0.05; the lines denote the fit of the empirical curves by the power curves of a one-sample *Z-test* of the mean (the standard deviations that produced the best fit are 0.056 for “FU + A” and 0.084 for “FU”). **(B)** The level of the test [shown as − log_10_(α) for better visibility] that yields power *w* = 0.8, as a function of the effect size in both methods (the curves are calculated using the abovementioned values of standard deviations). **(C)** Example of distributions of the estimates of the regression parameter (shown by histograms) for the scenario with γ = 0.4 in both methods. The lines show their fits by normal distributions (the Shapiro-Wilk *p*-values are 0.34 and 0.64 for “FU” and “FU + A,” respectively). The pentagram denotes the true value of the regression parameter.

We calculated the average values and standard deviations of parameter estimates in 1000 simulated datasets in both methods (i.e., with only follow-up data and with follow-up data and information on ages at biospecimen collection) for each γ. The results showed that the standard deviations of γ were smaller in the method that takes into account ages at biospecimen collection (0.057 vs. 0.085, in average; a 33.2% relative decrease). Although such a difference may look small in absolute value and not worth mentioning at first sight, it corresponds to a substantial gain in power, if we translate these observations into power curves and curves for the level of the test that yields specific power as a function of the effect size (regression parameter). Figure 1A illustrates the empirical power in these two methods (with follow-up only, “FU”, and follow-up and ages at biospecimen collection, “FU + A”) for different effect sizes (i.e., values of the parameter γ) and α = 0.05. We also fitted these empirical values with the power curves of a one-sample *Z-test* of the mean and found the values of the standard deviations that produced the best fit to the empirical power curves for each method (0.056 for “FU + A” and 0.084 for “FU”), see solid and dashed lines in Figure 1A. Figure 1B shows the level of the test [shown as − log_10_(α) for better visibility] that yields power *w* = 0.8, as a function of the effect size in both methods (the curves were calculated using the abovementioned values of standard deviations). Figure 1C shows an example of distributions of the estimates in simulated datasets in both methods and their fits by normal distributions (this example corresponds to γ = 0.4 for which the standard deviations and the relative decrease are close to the average ones: 0.058, 0.087, and 33.6%).

## Discussion

The results shown in Figure 1 indicate that the information on ages at biospecimen collection in addition to follow-up data gives a substantial increase in power compared to the traditional approach that uses the follow-up data only. It also follows from Figure 1 that for the effect size equal to 0.3 *p*-value reduces from 10^−2^ to 10^−5^ and for the effect size equal to 0.4 *p*-value drops from 10^−4^ to 10^−9^. This means that many genetic variants which would be not genome-wide significant in GWAS of follow-up data using the traditional Cox-type approach would become highly significant if the proposed approach is used. Our simulations with different follow-up periods (data not shown) reveal that this effect diminishes with an increase of a follow-up period. It is clear intuitively that, in the case of a growing follow-up period, information from this long follow-up makes an increasing contribution compared to information hidden in the distributions of ages at biospecimen collection. Conversely, in the case of a shorter follow-up period, distributions of ages at biospecimen collection play a more important role in differentiating the allele- or genotype-specific survival patterns from the data. Thus our results show that the additional use of information on ages at biospecimen collection may have important implications for GWA studies of longevity especially in cases with relatively short follow-up periods. The LLFS data provide a good example of a study which could potentially benefit from the addition of information on ages at biospecimen collection in its GWAS of longevity (as well as healthspan for which similar conclusions can be made). As longevity is known to run in families, family-based genome-wide association studies can provide additional advantages compared to analyses of independent samples. However, performing GWAS in cohort studies that contain both unrelated individuals and family members requires a special consideration of the analytical approach to take the full advantage of such data (Manichaikul et al., 2012).
